# Photon-counting *versus* energy-integrating CT of abdomen-pelvis: a phantom study on the potential for reducing iodine contrast media

**DOI:** 10.1186/s41747-025-00573-2

**Published:** 2025-03-23

**Authors:** Djamel Dabli, Maxime Pastor, Sebastian Faby, Julien Erath, Cédric Croisille, Fabricio Pereira, Jean-Paul Beregi, Joël Greffier

**Affiliations:** 1 Department of medical imaging, CHU Nîmes, Univ Montpellier, Nîmes Medical Imaging Group, UR UM103 IMAGINE, Nîmes, France; 2 Department of Computed Tomography, Siemens Healthineers AG, Forchheim, Germany; 3 MIPA, University of Nîmes, Nîmes, France

**Keywords:** Abdomen, Contrast media, Iodine compounds, Pelvis, Tomography (x-ray computed)

## Abstract

**Background:**

To assess the potential of virtual monoenergetic images (VMIs) on a photon-counting computed tomography (PCCT) for reducing the amount of injected iodine contrast media compared to an energy-integrating CT (EICT).

**Methods:**

A multienergy phantom was scanned with a PCCT and EICT at 11 mGy with abdomen-pelvis examination parameters. VMIs were generated at 40 keV, 50 keV, 60 keV, and 70 keV. For all VMIs, the contrast-to-noise ratio (CNR) of iodine inserts with concentrations of 1 mg/mL, 2 mg/mL, 5 mg/mL, 10 mg/mL, and 15 mg/mL was calculated by dividing the signal difference between HU in iodine inserts *versus* solid water by the noise value assessed on solid water. The potential reduction in iodine media was calculated by the rate of reduction in iodine concentration with PCCT while maintaining the same CNR obtained with EICT for the reference concentration.

**Results:**

Significantly higher CNR values were found with PCCT at all VMI energy levels for iodine concentrations above 1 mg/mL. The highest reduction was observed at 40 keV, with a value of 48.9 ± 1.6% (mean ± standard deviation). It decreased as the energy level increased, by 38.5 ± 0.5%, and 30.8 ± 0.8% for 50 and 60 keV, respectively. For 70 keV, the potential reduction of 24.4 ± 1.1% was found for iodine concentrations above 1 mg/mL. This reduction reached 57 ± 2.3% at 40 keV with PCCT compared to 60 keV with EICT.

**Conclusion:**

For abdomen-pelvis protocols, the use of VMIs with PCCT significantly improved the CNR of iodine, offering the potential to reduce the required contrast medium.

**Relevance statement:**

The use of VMIs with PCCT may reduce the quantity of iodine contrast medium to be injected compared with EICT, limiting costs, the risk of adverse effects, and the amount of contrast agent released into the wastewater.

**Key Points:**

- PCCT improves the image quality of VMIs.
- PCCT offers the potential for reducing the amount of injected contrast medium.
- PCCT potential for reducing the injected contrast medium depends on energy level.

**Graphical Abstract:**

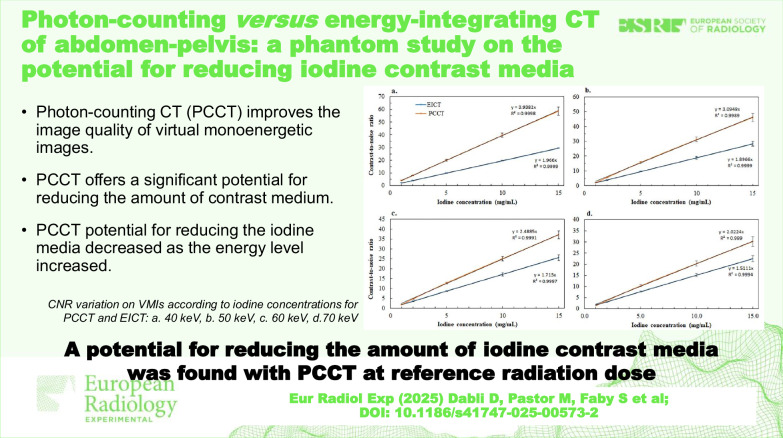

## Background

Computed tomography (CT) examinations are often combined with an injection of iodinated contrast media (ICM) to enhance the contrast of structures or lesions [1]. However, the risk of adverse effects from ICM must be taken into account in addition to the risk associated with x-ray exposure, especially in patients suffering from renal insufficiency [2–4].

The latest technological developments in CT acquisition and reconstruction methods [5–8] have made it possible to reduce both the radiation dose and the amount of ICM. Among these developments, dual-energy CT (DECT) equipped with energy-integrated detectors plays an important role. DECT utilizes two distinct spectra, characterized by lower and higher average energies compared to conventional CT, allowing differentiation of materials based on attenuation [9–13]. It is then possible to generate several types of images, notably, virtual monoenergetic images (VMIs). These images are visualized similarly to those that would have been obtained with a monochromatic beam which enhances the contrast of iodine in the tissues using low energy levels [14], improves the detection and characterization of abdominal lesions [14–18], and offers significant potential for reducing the amount of contrast media [19–21]. However, a phantom study by Van Hamersvelt et al [21] showed that iodine concentration could be reduced by up to 60% using VMIs at 40 keV on a dual-layer CT in angiography CT imaging of the aorta and coronary arteries.

Since 2021, a new generation of CT systems equipped with photon-counting detectors has become available. These new detectors are able to count photons according to their energy and have improved noise performance by suppressing electronic noise. They also have a better spatial resolution than energy-integrating detectors, thanks to their smaller detector elements [22–25]. Several studies have demonstrated a significant improvement in the image quality of VMIs with photon-counting CT (PCCT) and the potential of radiation dose reduction [26–31] compared to energy-integrating detector CT (EICT). Some studies have also demonstrated the potential of PCCT for reducing the amount of ICM estimated at 42% for pulmonary angiography CT [32], 40–50% for coronary CT angiography [33–35], and 25% for thoracoabdominal aorta CT angiography [36, 37]. However, these studies focused on chest and cardiac examinations. To the best of our knowledge, so far there have been no studies exploring the potential of VMIs generated with PCCT for reducing the amount of ICM injection in abdomen-pelvis examinations.

The reduction in the amount of ICM used needs to be consistent with maintaining sufficient image quality for diagnostic purposes. This means maintaining a sufficient contrast-to-noise ratio (CNR) for structures containing iodine. Several phantom studies have shown a linear relationship between CNR and the iodine content of structures for fixed mean beam energy [38, 39]. As a result, CNR can be used as a metric to assess the amount of contrast medium reduction possible while maintaining image quality similar to a reference image.

The purpose of this phantom study was to evaluate the potential of PCCT to reduce the amount of injected ICM compared to EICT, using DECT and VMI for abdomen-pelvis protocols, based on the CNR metric.

## Methods

### Phantom

**Fig. 1:**
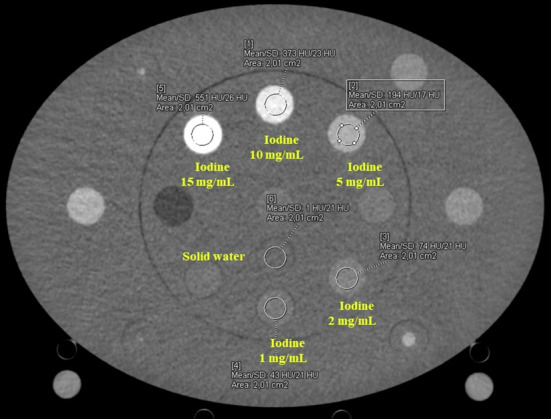
VMI at 50 keV with a region of interest placed in iodine and solid water inserts

Multienergy CT phantom commercialized by Sun Nuclear (Melbourne, Australia) of 20-cm diameter, with an elliptical ring (30 × 40 × 15 cm^3^) was scanned. The phantom is composed of a 20-cm diameter cylinder inserted on an elliptical ring allowing the phantom dimensions to be extended to represent a patient’s abdomen by inserting the 20-cm cylinder into the insert. The whole phantom with the elliptical insert measures 40 cm wide, 30 cm high, and 15 cm depth. The background material of the phantom is solid water. It contains 27 holes for solid inserts of 28 mm in diameter simulating different materials (Fig. 1).

Five iodine inserts with concentrations ranging from 1 mg/mL to 15 mg/mL and a solid water insert were placed in the center part of the phantom.

### Image acquisition and reconstruction

Acquisitions were performed on a PCCT (NAEOTOM Alpha, Siemens Healthineers, Forchheim, Germany) equipped with the photon-counting detector and on an EICT corresponding to the third-generation dual-source CT (SOMATOM Force, Siemens Healthineers). Two tube-detector pairs were available for PCCT and EICT. On the EICT, spectral acquisition is carried out using the two x-ray tubes with two different voltages, but on the PCCT this acquisition is carried out using a single x-ray tube [9].

Then, the default acquisition parameters for classical abdomen-pelvis DECT examinations were used on each scanner (Table 1). For EICT, 100/Sn150 kVp (tube A/tube B) was used, and 140 kVp for PCCT. For both scanners, the tube current value (mAs) was set to obtain a volume CT dose index of around 11 mGy, corresponding to the national guide value for abdomen-pelvis examinations. Five acquisitions were repeated for each CT scan.

**Table 1:** Acquisition and reconstruction parameters used on energy-integrated and PCCT

Scanner model	SOMATOM Force	NAEOTOM Alpha
Detector	Stellar (EID)	QuantaMax (PCD)
Collimation (mm)	128 × 0.6 mm	144 × 0.4 mm
Pitch	0.8	0.8
Rotation time (s)	0.5	0.5
Tube voltage (kV)	100/Sn150	140
Displayed CTDI_vol_ (mGy)	10.96	11.00
Tube current (mAs) (tube A/tube B for EID and tube A for PCD)	189/95	96
Reconstruction kernel	Qr36	Qr36
Reconstruction algorithm	ADMIRE	QIR
Level	3	3

*ADMIRE* Advanced modeled iterative reconstruction, *CTDI*_*vol*_ Volume CT dose index, *EID* Energy-integrated, *PCD* Photon-counting, *QIR* Quantum iterative reconstruction

Raw data were reconstructed for both CT scans using the soft tissue quantification kernel (Qr36), a slice thickness of 1 mm (in 1-mm increments), and a 420-mm field of view. Level 3 of the Advanced Modeled Iterative Reconstruction algorithm was used for EICT and level 3 of Quantum Iterative Reconstruction algorithm for PCCT.

For all CT acquisitions, VMIs were generated at four low energy levels (40 keV, 50 keV, 60 keV, and 70 keV) using the “Monoenergetic+” application of the *syngo.via* postprocessing software (version VB60A_HF03, Siemens Healthineers).

### CNR measurement

Several studies have reported that ICM concentration values in abdominal structures ranged from 5 mg/mL to 10 mg/mL in the aorta [40–42] and 1.23–4.31 mg/mL in other abdominal organs or lesions [43–46]. Thus, iodine inserts with concentrations of 1 mg/mL, 2 mg/mL, 5 mg/mL, 10 mg/mL, and 15 mg/mL were used in this study to be representative of clinical concentrations. The solid water insert was also used as a reference representing tissues not containing iodine. For each energy level, six circular regions of interest (ROIs) of 2 cm² were placed in the center of the solid water insert and on five iodine inserts on the central slice of the phantom (Fig. 1). For each ROI, the mean HU value was measured on each VMI using the “Monoenergetic +” application in the syngo.via software.

The CNR was defined as the signal difference between HU in iodine inserts *versus* background (solid water), divided by background noise. For each iodine insert, the iodine CNR was calculated using the following Eq. 1:

1 $${{{\rm{CNR}}}}=\frac{|\,{{{\rm{HU}}}}_{{{\rm{ROI}}}}-{{{\rm{HU}}}}_{{{\rm{SW}}}}|}{{{{\rm{SD}}}}_{{{\rm{SW}}}}}$$

where HU_ROI_ corresponds to the HU value measured in a ROI placed in the center of one iodine insert. HU_SW_ corresponds to the HU value measured in the ROI of the solid water insert and SD_SW_ is the standard deviation in the same ROI representing the image noise.

For each CT system, contrast, image noise, and CNR values were measured for the four energy levels of VMIs and the five acquisitions. The values obtained for each metric for both CT systems were then compared.

### Potential ICM amount reduction

The variation in CNR according to iodine concentration was determined for VMIs generated by PCCT and EICT at four energy levels.

Assuming the proportional correlation between CNR and the iodine delivery rate as reported by Domenico De Santis et al [38], the potential reduction in iodine was calculated by the rate of reduction in tissue iodine concentration with PCCT while maintaining the same CNR obtained with EICT for the reference concentration. This is illustrated by the following Eq. 2:

2 $${{{{\rm{Potential}}}}\;{{{\rm{iodine}}}}\;{{{\rm{reduction}}}}}\;(\%)=\frac{\,{C}_{{{\rm{PCCT}}}}-{C}_{{{\rm{EICT}}}}}{{C}_{{{\rm{EICT}}}}}\times 100$$

Where ${C}_{{{\rm{PCCT}}}}$ are the iodine concentration required with the PCCT to obtain the same CNR value as that measured on the EICT with the reference concentration noted by ${C}_{{{\rm{EICT}}}}$.

### Statistical analyses

Statistical analyses were performed using version 3.5.1 of R software (R Core Team, 2017, Vienna, Austria). The Shapiro–Wilk test was used to verify the normality of data distribution for HU, noise, and CNR values at different energy levels. As all data were normally distributed (*p*-values > 0.05). Contrast, noise, and CNR values were compared between PCCT and EICT VMIs using the Student *t*-test for all four energy levels. A *p*-value ≤ 0.050 was considered significant.

## Results

### CNR measurement

Table 2 shows the contrast values of each iodine insert according to the energy level for both scanners. HU values for iodine inserts and solid water are shown in Table S1 of the supplementary material. For all energy levels and concentrations ranging from 2 mg/mL to 15 mg/mL, no significant difference was observed between the two CT scanners (*p* > 0.05; Table 2). For 1 mg/mL and all energy levels, contrast values were significantly lower with PCCT with a mean difference of 38.8 ± 3.9% (mean ± standard deviation).

**Table 2:** Contrast values between iodine inserts and solid water insert according to monoenergetic levels for EICT and PCCT

Inserts	Energy level (keV)	EICT	PCCT	*p*-value
Iodine at 15 mg/mL	40	1234.2 ± 8.2	1242.0 ± 5.9	0.091
	50	807.4 ± 5.4	813.1 ± 3.5	0.069
	60	548.3 ± 3.9	552.1 ± 1.8	0.056
	70	388.5 ± 3.1	389.0 ± 4.6	0.855
Iodine at 10 mg/mL	40	820.7 ± 9.4	839.5 ± 5.9	0.057
	50	538.5 ± 6.1	549.5 ± 3.2	0.061
	60	367.2 ± 4.1	372.9 ± 1.4	0.052
	70	261.6 ± 3.0	262.0 ± 4.5	0.892
Iodine at 5 mg/mL	40	416.3 ± 8.9	422.9 ± 4.5	0.206
	50	273.3 ± 6.1	277.6 ± 2.3	0.237
	60	186.7 ± 4.5	189.3 ± 1.0	0.274
	70	133.3 ± 3.5	133.0 ± 4.6	0.906
Iodine at 2 mg/mL	40	172.0 ± 11.7	147.5 ± 4.7	0.052
	50	111.8 ± 7.1	98.9 ± 3.0	0.056
	60	75.7 ± 3.8	69.0 ± 1.9	0.063
	70	53.4 ± 2.3	48.3 ± 5.5	0.136
Iodine at 1 mg/mL	40	87.9 ± 11.8	54.5 ± 5.8	**0.009**
	50	59.0 ± 8.0	36.9 ± 2.9	**0.007**
	60	42.2 ± 5.0	26.1 ± 1.3	**0.001**
	70	31.9 ± 3.2	17.6 ± 5.0	**0.004**

Significant *p*-values are shown in bold

**Fig. 2:**
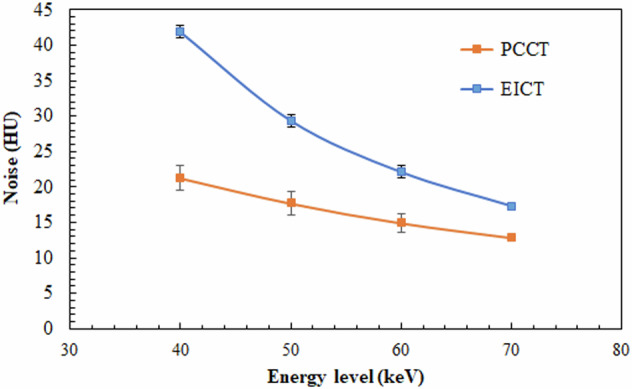
Noise variation on VMIs according to energy level for PCCT and energy-integrating computed tomography (EICT) at a reference radiation dose of 11 mGy

For both scanners, the noise values decreased significantly as the energy level decreased (Fig. 2). For all energy levels, the noise values were significantly lower for PCCT with a mean difference of (49.2 ± 4.3) % at 40 keV, (39.8 ± 5.1)% at 50 keV, (32.5 ± 5.8)% at 60 keV and (25.4 ± 4.0)% at 70 keV.

**Fig. 3:**
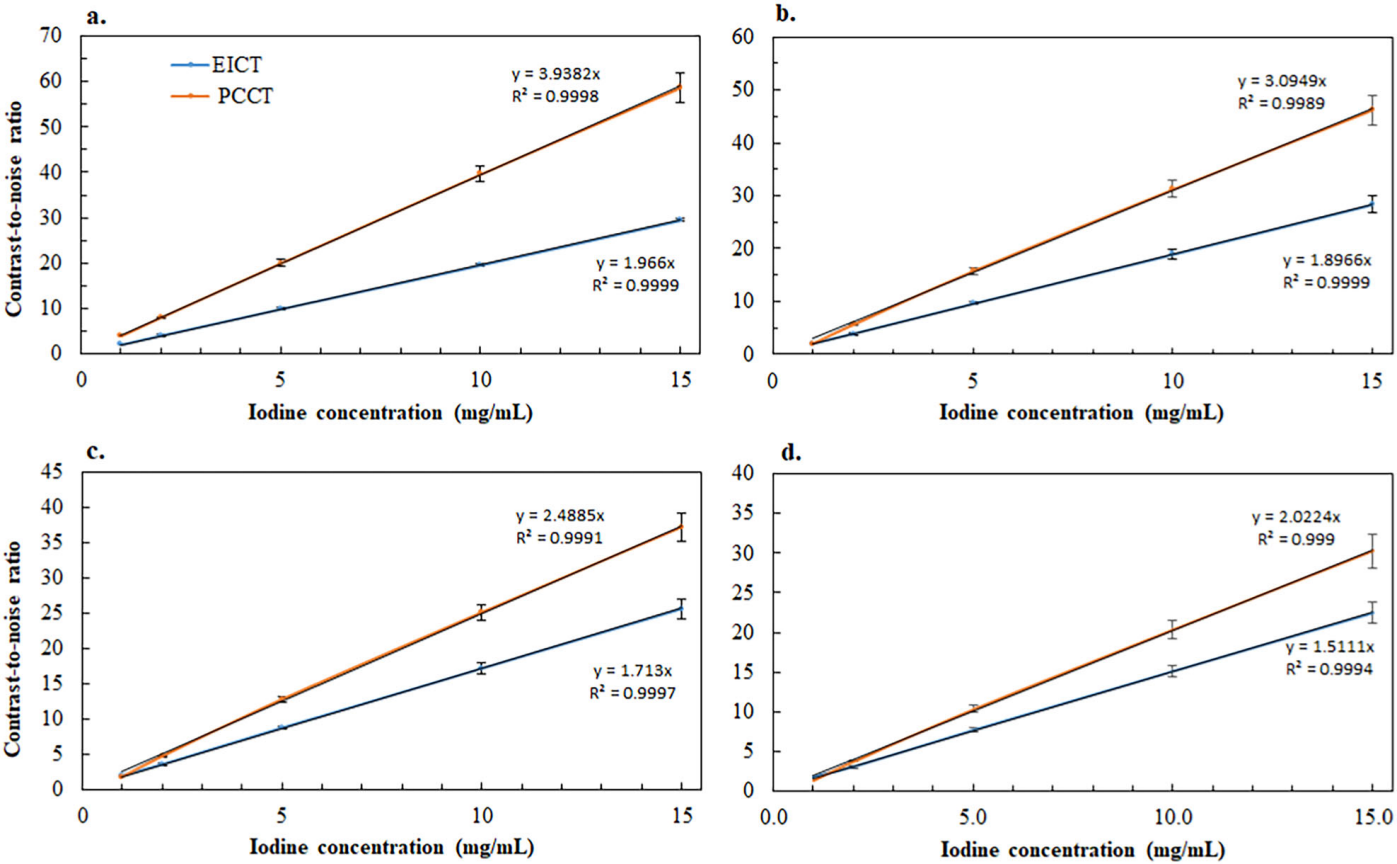
CNR variation on VMIs according to iodine concentrations for PCCT and EICT: **a** CNR variation for 40 keV energy level; **b** CNR variation for 50 keV energy level; **c** CNR variation for 60 keV energy level; **d** CNR variation for 70 keV energy level. CNR, Contrast-to-noise ratio

For both scanners, the CNR values increased significantly as the iodine concentration increased (Fig. 3). For both CT scanners, the CNR values increased significantly as the energy level decreased for iodine concentrations ranging from 2 mg/mL to 15 mg/mL. The opposite pattern was found at 1 mg/mL, except between 60 keV and 70 keV (*p* = 0.066). For iodine concentrations ranging from 5 mg/mL to 15 mg/mL, the mean CNR values were significantly higher with PCCT than with EICT, at all energy levels (*p* < 0.001). The difference between PCCT and EICT decreased as energy levels increased by (101.6 ± 1.6) % at 40 keV, (69.6 ± 1.1) % at 50 keV, (50.6 ± 0.7) % at 60 keV, and (34.7 ± 0.4) % at 70 keV.

With iodine concentration of 2 mg/mL, the mean CNR values were significantly higher with PCCT than with EICT only at energy levels of 40 keV (70.5 ± 18.5%, *p* = 0.002), 50 keV (48.3 ± 17.8%, *p* = 0.020), and 60 keV (36.1 ± 16.5%, *p* = 0.040).

For 1 mg/mL and all energy levels, higher CNR values were found with PCCT than with EIDCT but the differences were not significant with *p*-values of 0.206, 0.798, 0.606, and 0.356 for 40 keV, 50 keV, 60 keV, and 70 keV, respectively.

### Potential ICM reduction

Table 3 shows the potential iodine reduction rate according to iodine concentration and energy levels for PCCT, compared with the standard EICT acquisition whilst maintaining the same CNR.

**Table 3:** Potential iodine quantity reduction rate according to iodine concentration and energy level for photon counting CT (PCCT) compared with the energy integrating CT (EICT) while maintaining the same CNR

Iodine concentration (mg/mL)	40 keV	50 keV	60 keV	70 keV
1	47%	33%	21%	9%
2	48%	37%	29%	24%
5	49%	38%	30%	24%
10	50%	39%	31%	25%
15	50%	39%	31%	26%

The highest reduction potential was found at 40 keV for all iodine concentrations with a mean value of 48.9 ± 1.6%. Potential ICM reduction decreased as the energy level increased, with mean values of 38.5 ± 0.5%, 30.8 ± 0.8% for 50 keV and 60 keV, respectively. For 70 keV, the mean potential reduction of 24.4 ± 1.1% was found for iodine concentrations over 1 mg/mL and a non-significant potential reduction was found for 1 mg/mL.

## Discussion

This study demonstrated that for abdomen-pelvis examinations, PCCT provided a lower noise level on VMIs, compared to EICT images. The CNR values were significantly higher with PCCT for all energy levels and iodine concentrations except for 1 mg/mL concentration for which the difference was not significant. A potential iodine reduction rate was found for all iodine concentrations and energy levels except for 1 mg/mL at 70 keV. This potential iodine reduction decreased as the energy level increased.

The results showed an improvement in CNR values with PCCT VMIs compared to EICT. These results are explained by the PCCT’s performance in terms of noise reduction on VMIs [22, 45]. In fact, the noise results measured on VMIs showed that the PCCT achieved a reduction ranging from (49.2 ± 4.3) % to (25.4 ± 4.0) % for energy levels from 40 keV to 70 keV, respectively. Further, the CNR values increased linearly with increasing iodine concentration for all energy levels and CT scanners. Similar results were found by De Santis et al [38] and Kok et al [39] on phantoms. Assuming that this linear relationship is also valid for patient images, the improvement in CNR can be exploited to reduce the amount of contrast media with PCCT while maintaining a CNR similar to that obtained with EICT.

For the same energy level of VMIs, the potential reduction in the amount of contrast media was found similar whatever the initial concentration, except for 1 mg/mL at 70 keV, where this potential was lower than for other concentrations. However, this potential decreased as the monoenergetic level of VMIs increased. It should be noted that the concentrations reported in the literature in the various abdominal structures are higher than 1 mg/mL, with concentrations ranging from 5 mg/mL to 10 mg/mL in the aorta [41, 42] and from 1.23 mg/mL to 4.31 mg/mL in upper abdominal structures [6] or from 1.32 ± 0.33 mg/mL to 2.03 ± 0.42 mg/mL in hepatocellular lesions [43, 44], according to the injection phase (arterial or portal). It is well known that, the monoenergetic level used to interpret VMI for abdominal examinations ranges from 40 keV to 60 keV [15, 46]. Therefore, the potential reduction in the amount of ICM expected for these examinations if the same energy level was used with EICT and PCCT would range from 49 ± 1% at 40 keV to 28 ± 4% at 60 keV energy level. However, the improvement in CNR of VMIs at low energy levels with PCCT offers the possibility of using a lower energy level than with EICT for abdomen imaging. In this case, the VMIs obtained with PCCT offer a higher reduction potential than when using the same energy level as with EICT. This reduction reached 57 ± 2.3% if the energy level used with EICT was 60 keV and that with PCCT was 40 keV.

Transposing these results into clinical application requires vigilance in adapting the injection rate to ensure the injection time is long enough. The injection rate can be calculated from the volume to be injected and the injection time to be respected [47]. The purpose of this adaptation is to maintain a similar distribution of contrast medium in the tissues between acquisition with PCCT and the reference acquisition with EICT. Finally, the results of this study suggest that the improvement in image quality of the VMIs produced by PCCT offers significant prospects to optimize the amount of contrast media injected into patients during abdominal examinations and the amount of contrast agent released into the wastewater, in the years to come.

This study has certain limitations. The first obvious one is that it was a phantom study, limiting the generalizability of results to patients. The CNR values were measured on a phantom with uniform iodine inserts rather than on actual structures of a human abdomen. These results must therefore be confirmed by a study on real patient images. The second limitation concerns the position of the iodine inserts in the center of the phantom; results may differ at the periphery of the phantom. The third is that these acquisition parameters were explored based on our clinical experience with the EICT scanner and the manufacturer’s recommended parameters for the PCCT scanner. Finally, the iodine reduction potential is calculated on the basis of a linear relationship between the iodine concentration in the inserts and the CNR. This relationship is demonstrated on phantom, but needs to be verified on patient images by measuring tissue concentration and associated CNR.

In conclusion, for abdomen-pelvis CT protocols, this phantom study showed that using VMIs with PCCT significantly improves the CNR of iodine compared to EICT, offering substantial potential to reduce the amount of required ICM.

## Supplementary information

**Additional file 1: Table 1 SM:** Mean HU values over a 5 repeated acquisition and associated standard deviation measured for iodine and solid water compared to theoretical values for virtual monochromatic images (VMIs) according to four energy levels for photon-counting CT and energy-integrating CT at standard radiation dose of 11 mGy. **Table 2 SM:** Mean contrast-to-noise (CNR) values over a 5 repeated acquisition and associated standard deviation measured for iodine inserts in virtual monochromatic images (VMIs) according to four energy levels for photon-counting CT and energy-integrating CT at standard radiation dose of 11 mGy.

## Data Availability

The datasets analyzed during the current study are available from the corresponding author upon reasonable request.

## Abbreviations

CNR: Contrast-to-noise ratio
CT: Computed tomography
DECT: Dual-energy CT
EICT: Energy-integrating CT
ICM: Iodine contrast media
PCCT: Photon-counting CT
ROI: Region of interest
VMI: Virtual monoenergetic image
