# Supplementary material for: Photon-counting versus energy-integrating CT of abdomen-pelvis: a phantom study on the potential for reducing iodine contrast media
Source: Eur Radiol Exp. 2025 Mar 23;9:36. doi: 10.1186/s41747-025-00573-2 (PMC11930902; doi:10.1186/s41747-025-00573-2)
Supplement: Supplementary file 1 — Additional file 1: Table 1 SM: Mean HU values over a 5 repeated acquisition and associated standard deviation measured for iodine and solid water compared to theoretical values for virtual monochromatic images (VMIs) according to four energy levels for photon-counting CT and energy-integrating CT at standard radiation dose of 11 mGy. Table 2 SM: Mean contrast-to-noise (CNR) values over a 5 repeated acquisition and associated standard deviation measured for iodine inserts in virtual monochromatic images (VMIs) according to four energy levels for photon-counting CT and energy-integrating CT at standard radiation dose of 11 mGy. [file 41747_2025_573_MOESM1_ESM.pdf]

Photon-counting versus energy-integrating CT of abdomen-pelvis: a phantom study on the potential for reducing iodine contrast media

ELECTRONIC SUPPLEMENTARY MATERIAL

Table 1 SM: Mean HU values over a 5 repeated acquisition and associated standard deviation measured for iodine and solid water compared to theoretical values for virtual monochromatic images (VMIs) according to four energy levels for photon-counting CT and energy-integrating CT at standard radiation dose of 11 mGy.

Inserts	Energy level (keV)	Theoretical HU value	EICT	PCCT
Iodine at 15 mg/mL	40	1265	1232.64 ± 7.91	1279.66 ± 4.63
	50	833	806.74 ± 5.05	833.72 ± 2.97
	60	564	547.67 ± 3.29	562.37 ± 1.8
	70	397	387.92 ± 2.24	394.93 ± 0.84
Iodine at 10 mg/mL	40	843	819.07 ± 6.28	877.22 ± 3.49
	50	555	537.74 ± 4.20	570.13 ± 2.13
	60	375	366.60 ± 2.93	383.23 ± 1.28
	70	263	261.11 ± 2.16	267.89 ± 0.81
Iodine at 5 mg/mL	40	426	414.70 ± 10.79	460.55 ± 3.27
	50	280	272.56 ± 6.87	298.26 ± 2.09
	60	189	186.12 ± 4.50	199.58 ± 1.27
	70	133	132.81 ± 3.12	138.88 ± 0.87
Iodine at 2 mg/mL	40	171	170.44 ± 7.49	185.21 ± 4.68
	50	112	111.04 ± 4.29	119.49 ± 2.98
	60	75	75.09 ± 2.31	79.26 ± 1.9
	70	52	52.93 ± 1.25	54.17 ± 1.06
Iodine at 1 mg/mL	40	88	86.33 ± 6.58	92.22 ± 3.94
	50	58	58.30 ± 4.66	57.54 ± 2.09
	60	38	41.64 ± 3.20	36.42 ± 1.07
	70	26	31.36 ± 2.36	23.5 ± 0.80
Iodine at 0.5 mg/mL	40	46	42.61 ± 5.20	56.68 ± 2.17
	50	30	27.39 ± 4.67	35.53 ± 1.74
	60	19	19.67 ± 3.06	22.28 ± 1.07
	70	13	14.93 ± 2.13	13.53 ± 0.96
Solid water	40	-2	-1.6 ± 5.69	37.7 ± 3.29
	50	-3	-0.74 ± 3.82	20.63 ± 1.55
	60	-4	-0.61 ± 2.29	10.3 ± 0.51
	70	-5	-0.52 ± 1.60	5.92 ± 4.68

No significant difference was observed in HU values of PCCT and EICT for all iodine inserts and all energy levels (p>0.05). For solid water insert significant higher values were found wit PCCT for all energy levels (p value ranged from 0.001 and 0.02).

Table 2 SM: Mean contrast-to-noise (CNR) values over a 5 repeated acquisition and associated standard deviation measured for iodine inserts in virtual monochromatic images (VMIs) according to four energy levels for photon-counting CT and energy-integrating CT at standard radiation dose of 11 mGy.

Energy level (keV)	Iodine cocentration (mg/mL)	EICT	PCCT
40	1	2.1 ± 0.3	3.9 ± 0.2
	2	4.1 ± 0.3	8.0 ± 0.7
	5	9.9 ± 0.3	20.0 ± 1.8
	10	19.6 ± 0.4	39.7 ± 3.2
	15	29.5 ± 0.4	58.7 ± 5.1
50	1	2.1 ± 0.2	2.1 ± 0.2
	2	3.9 ± 0.3	5.6 ± 0.7
	5	9.6 ± 0.8	15.8 ± 1.6
	10	18.9 ± 1.5	31.3 ± 2.8
	15	28.4 ± 2.3	46.3 ± 4.4
60	1	2.0 ± 0.2	1.8 ± 0.1
	2	3.5 ± 0.2	4.6 ± 0.5
	5	8.7 ± 0.7	12.7 ± 1.1
	10	17.1 ± 1.4	25.1 ± 2.1
	15	25.6 ± 2.1	37.2 ± 3.2
70	1	1.8 ± 0.1	1.4 ± 0.4
	2	3.1 ± 0.1	3.7 ± 0.4
	5	7.7 ± 0.6	10.3 ± 0.4
	10	15.2 ± 1.2	20.4 ± 0.7
	15	22.5 ± 1.8	30.2 ± 1
